# Multiple controls affect arsenite oxidase gene expression in *Herminiimonas arsenicoxydans*

**DOI:** 10.1186/1471-2180-10-53

**Published:** 2010-02-18

**Authors:** Sandrine Koechler, Jessica Cleiss-Arnold, Caroline Proux, Odile Sismeiro, Marie-Agnès Dillies, Florence Goulhen-Chollet, Florence Hommais, Didier Lièvremont, Florence Arsène-Ploetze, Jean-Yves Coppée, Philippe N Bertin

**Affiliations:** 1 UMR7156 Génétique Moléculaire, Génomique et Microbiologie, CNRS Université de Strasbourg, 28 rue Goethe, 67000 Strasbourg, France; 2 Plate-forme technologique Puces à ADN, Institut Pasteur, 28 rue du Dr. Roux, 75724 Paris cedex 15, France; 3 UMR5240 Microbiologie, Adaptation et Pathogénie, CNRS Université Lyon 1, Bâtiment André Lwoff, 10 rue Dubois, 69622 Villeurbanne cedex France

## Abstract

**Background:**

Both the speciation and toxicity of arsenic are affected by bacterial transformations, i.e. oxidation, reduction or methylation. These transformations have a major impact on environmental contamination and more particularly on arsenic contamination of drinking water. *Herminiimonas arsenicoxydans *has been isolated from an arsenic- contaminated environment and has developed various mechanisms for coping with arsenic, including the oxidation of As(III) to As(V) as a detoxification mechanism.

**Results:**

In the present study, a differential transcriptome analysis was used to identify genes, including arsenite oxidase encoding genes, involved in the response of *H. arsenicoxydans *to As(III). To get insight into the molecular mechanisms of this enzyme activity, a Tn*5 *transposon mutagenesis was performed. Transposon insertions resulting in a lack of arsenite oxidase activity disrupted *aoxR *and *aoxS *genes, showing that the *aox *operon transcription is regulated by the AoxRS two-component system. Remarkably, transposon insertions were also identified in *rpoN *coding for the alternative N sigma factor (σ^54^) of RNA polymerase and in *dnaJ *coding for the Hsp70 co-chaperone. Western blotting with anti-AoxB antibodies and quantitative RT-PCR experiments allowed us to demonstrate that the *rpoN *and *dnaJ *gene products are involved in the control of arsenite oxidase gene expression. Finally, the transcriptional start site of the *aoxAB *operon was determined using rapid amplification of cDNA ends (RACE) and a putative -12/-24 σ^54^-dependent promoter motif was identified upstream of *aoxAB *coding sequences.

**Conclusion:**

These results reveal the existence of novel molecular regulatory processes governing arsenite oxidase expression in *H. arsenicoxydans*. These data are summarized in a model that functionally integrates arsenite oxidation in the adaptive response to As(III) in this microorganism.

## Background

Arsenic is present in various environments, released from either anthropogenic or natural sources. This element is toxic for living organisms and known to be a human carcinogen [1]. Its toxicological effects depend, at least in part, on its oxidation state and its chemical forms, inorganic species being considered as more toxic [2]. The contamination of drinking water by the two inorganic forms, arsenite As(III) and arsenate As(V), has been reported in different parts of the world [3] and constitutes a major threat of public health. Microorganisms are known to take part in the transformation, i.e oxidation, reduction or methylation of the metalloid, having a deep impact on arsenic contamination in environment.

Several bacteria and prokaryotes have developed adaptation, resistance and colonization mechanisms, which allow them to live in hostile arsenic contaminated environments. *H. arsenicoxydans *is a Gram-negative β-proteobacterium isolated from an industrial activated sludge plant and exhibiting a remarkable set of arsenic resistance determinants [4]. The *H. arsenicoxydans *adaptive response to arsenic is organized in a complex and sophisticated network. In particular, differential proteome studies have recently demonstrated the synthesis of several proteins encoded by the three *ars *resistance operons, e.g. arsenate reductase ArsC, flavoprotein ArsH and regulator ArsR [5,6] and the induction of oxidative stress protein encoding genes, e.g. catalase (*katA*), superoxide dismutase (*sodB*) and alkyl hydroperoxide reductase (*ahpC*) [7].

One of the most noticeable response to arsenic in *H. arsenicoxydans *is the ability of this bacterium to oxidize As(III) to As(V), a less toxic and less mobile form, *via *an arsenite oxidase activity. The two genes coding for this heterodimeric enzyme are organized in an operonic structure, and have been named *aoxA *and *aoxB *for the small and the large subunit, respectively [6,8,9]. Homologous genes have been since identified in various microorganisms [6,10-13]. In *Agrobacterium tumefaciens*, a complex transcriptional regulation has been recently suggested, involving As(III) sensing, two-component signal transduction by an AoxS sensor kinase and an AoxR regulator, and quorum sensing [14]. Nevertheless, the molecular mechanisms involved in the control of arsenite oxidase expression remain largely unknown.

To extend our view of arsenic response [7], in particular with respect to the arsenic oxidation process, we conducted a comprehensive transcriptomic, genetic and molecular analysis of *H. arsenicoxydans *following exposure to As(III). These approaches allowed us to identify major determinants involved in the control of arsenite oxidation.

## Results

### Gene expression profiling in response to arsenic

The response to As(III) was analyzed in exponentially growing cells using microarrays. The data from three biological replicates were combined after normalization and statistical analysis carried out using the R software and packages http://www.r-project.org. The set of genes was further refined to include only those genes that showed a valid *p*-value and whose expression was altered by a factor of 2 or more when compared to the level measured in the absence of arsenic.

This experiment led to the identification of 293 genes showing an arsenic-induced expression change (> 2 fold (log_2 _= 1)). Among these genes, 133 (45%) were up-regulated and the remaining part, i.e. 160 genes, was down-regulated. The relative changes in gene expression ranged from a 11-fold down-regulation (*rpsN *gene encoding a ribosomal protein) to a 126-fold up-regulation (putative gene involved in phosphate transport). A list of those genes is shown in Additional file 1, Table S1. The corresponding HEAR gene numbers are available in the Arsenoscope relational database http://www.genoscope.cns.fr/agc/mage/arsenoscope*via *the MaGe web interface [15].

The 293 genes differentially expressed were clustered according to the function of the corresponding encoded proteins. Among the 133 genes that were induced by at least a 2-fold factor, about 11% are involved in metabolism, 26% in transport and binding protein, 26% in cellular processes and 31% have no assigned function. The high percentage of genes with unknown function is in accordance with the proportion of unknown function proteins identified in the genome of *H. arsenicoxydans *[6,7]. In agreement with our previous results, genes involved in arsenic resistance, phosphate transport and flagellar biosynthesis were induced in the presence of As(III) (see Additional file 1, Table S1), further supporting the relationship between these physiological processes [6,7]. Interestingly, only one methyl-accepting chemotaxis protein (MCP) gene was induced in our microarray data, suggesting a role for this protein in the sensing of arsenic. This mechanism is currently under investigation. Genes encoding the putative nitroreductase AoxC and the cytochrome c_552 _precursor AoxD as well as the response regulator AoxRS were found to be induced by As(III) (see Additional file 1, Table S1). AoxR has been proposed to act as a positive regulator of the *aox *operon upon phosphorylation by AoxS in *A. tumefaciens *[14]. Our transcriptomic data suggest that the regulation machinery is, at least in part, similar in *H. arsenicoxydans*. Futhermore, genes coding for the arsenite oxidase AoxAB subunits were found to be among the most induced genes in the presence of As(III). However, even though these results extended our knowledge of the arsenic response in *H. arsenicoxydans*, they did not led to a better understanding of the molecular mechanisms involved in the control of arsenite oxidation. This prompted us to perform a transposon mutagenesis experiment.

### Identification of arsenite oxidase accessory genes by screening an Aox activity deficient mutant library

**Figure 1:**
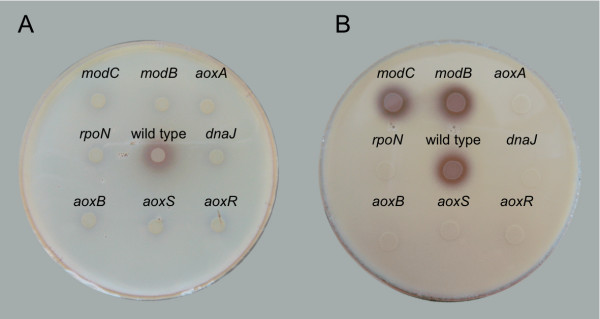
**Effect of the various mutations on arsenite oxidase activity**. This reaction was tested on plate after silver nitrate staining. Colonies expressing arsenite oxidase activity revealed a brownish precipitate on CDM solid medium. A. Detection of mutants without arsenite oxidase activity after 48 hours incubation on CDM plates. B. Recover of arsenite oxidase activity in *modB *and *modC *mutants in the presence of 50 μM Mo in the solid CDM medium.

**Figure 2:**
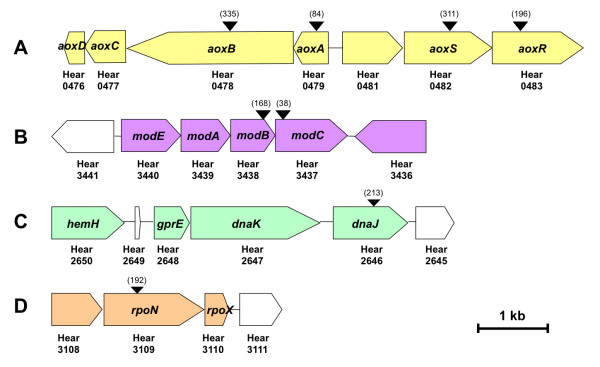
**Genomic organization of the chromosomal regions (A, B, C and D) containing genes involved in arsenite oxidase activity**. Genes orientation is shown by arrows. The insertion positions of the mini-Tn*5*::*lacZ2 *transposon are indicated above the mutated genes by inverted arrowheads. The position of the codon immediately upstream of the transposon insertion site is indicated in brackets.

To identify genes possibly involved in the control of arsenite oxidation in *H. arsenicoxydans*, a library of 10,000 kanamycin resistant mutants was constructed by transposon mutagenesis, as previously described [9]. These clones were tested by silver nitrate staining [16] for arsenate production on As(III)-supplemented CDM agar plates. As compared to the wild-type strain, whose arsenite oxidase activity was revealed by a brownish precipitate, 10 mutants with a lack of As(III) oxidase activity were obtained. These strains showed no precipitate (Figure 1A), as observed for the M1 and M2 strains used as negative controls. Indeed, these strains carry a mutation in *aoxA *or *aoxB *genes coding for the small and the large subunit of arsenite oxidase, respectively [9]. Genes disrupted by transposon insertions were identified in these 10 new mutants. As expected, four of the 10 mutants showed insertions in the *aoxAB *operon (Figure 2A). More interestingly, six mutants carried a transposon insertion outside the *aoxAB *operon. Two mutants were found to be affected in the *aoxRS *two-component signal transduction system (mutants Ha482 and Ha483, respectively) located upstream of the *aoxAB *operon in *H. arsenicoxydans *[6] (Figure 2A). These results further support our transcriptomic data suggesting that these two genes play a role in arsenic response. Two transposon insertions were shown to disrupt genes of the *modEABC *operon coding for a molybdenum high-affinity transport system [17], i.e. *modC *encoding an ATP-binding cassette transport protein (mutant Ha3437) and *modB *encoding a molybdenum transport system permease (mutant Ha3438) (Figure 2B). Remarkably, transposon insertions were also located in *dnaJ *encoding a heat shock protein (Hsp40), (mutant Ha2646) (Figure 2C) and in *rpoN *encoding the alternative nitrogen sigma factor (sigma 54) of RNA polymerase (mutant Ha3109) (Figure 2D).

Two additional experiments were performed to complete the physiological characterization of these mutants with respect to arsenite oxidation. First, arsenic species were quantified by HPLC-ICP-AES on filtered culture supernatants. *H. arsenicoxydans *was grown in liquid medium supplemented with 1.33 mM arsenite and showed 100% transformation of As(III) into As(V) after 48 h, whereas M1 (*aoxA*) and M2 (*aoxB*) mutants used as controls were not able to transform As(III) into As(V). The same loss of arsenite oxidase activity was measured in Ha482 (*aoxS*), Ha483 (*aoxR*), Ha2646 (*dnaJ*) and Ha3109 (r*poN*) mutants. In contrast to the results obtained on agar plates, Ha3437 (*modC*) and Ha3438 (*modB*) strains showed 100% transformation of arsenite (Table 1, Figure 1A). Previous studies have demonstrated that the bioavailability of metals or trace elements considerably varies according to the type of matrix used for microbial growth [18]. We therefore assumed that Mo might be partly sequestred on CDM agar medium, resulting in a lack of arsenite oxidase activity on plate. To test this hypothesis, As(III) oxidase tests were performed on CDM agar plates supplemented with 50 μM Mo. The addition of Mo to the solid medium restored As(III) oxidase activity in both Ha3437 (*modC*) and Ha3438 (*modB*) mutants while it had no effect on other mutant strains (Figure 1B).

**Table 1:** Determination of arsenic speciation in *H. arsenicoxydans *wild-type and mutant strains.

Strain	Mutated gene	**Arsenic species identified**^**a**^	
		**As(III)**	**As(V)**

ULPAs1	/	-	+
M1^b^	*aoxA*	+	-
M2^b^	*aoxB*	+	-
Ha482	*aoxS*	+	-
Ha483	*aoxR*	+	-
Ha2646	*dnaJ*	+	-
Ha3109	*rpoN*	+	-
Ha3437	*modC*	-	+
Ha3438	*modB*	-	+

Determined by HPLC-ICP-AES after 48 h growth in CDM medium containing 100 mg.liter of arsenite.

^b ^[9]

**Figure 3:**
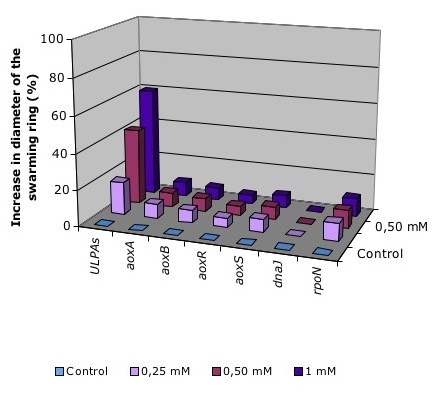
**Effect of arsenite concentration on swarming properties in *H. arsenicoxydans *wild-type and mutant strains**. Motility assays were performed in the presence of an increased concentration of As(III). The level of motility of each strain was evaluated as the diameter of the swarming ring expressed in mm. The results are the mean value of five independent experiments.

Second, we have previously demonstrated that the polar flagellum-dependent motility of *H. arsenicoxydans *is increased in the presence of As(III), suggesting that arsenite oxidation may result in a gain of energy [6]. The motility of mutant strains was therefore tested on plates containing different concentrations of As(III), i. e. 0.66 mM, 1.33 mM and 2 mM. The diameter of the swarming rings was measured after 72 h. As shown in Figure 3, the disruption of *aoxA, aoxB, aoxR, aoxS *or *rpoN *genes abolished the improvement of swarming performances in the presence of As(III). Unlike those mutants, a disruption in *dnaJ *completely abolished the motility of *H. arsenicoxydans *in the presence or the absence of As(III). DnaJ is known to be essential for the expression of the *flhDC *flagellar master operon in *Escherichia coli *[19]. The lack of motility observed in the *dnaJ *mutant suggests the existence of a similar *flhDC*-dependent regulation of flagellar genes in *H. arsenicoxydans*. More importantly, the *dnaJ *mutant was affected in both the motility and arsenite oxidation, suggesting that these two mechanisms are co-regulated.

### Effect of AoxR, AoxS, RpoN and DnaJ on arsenite oxidase synthesis

**Figure 4:**
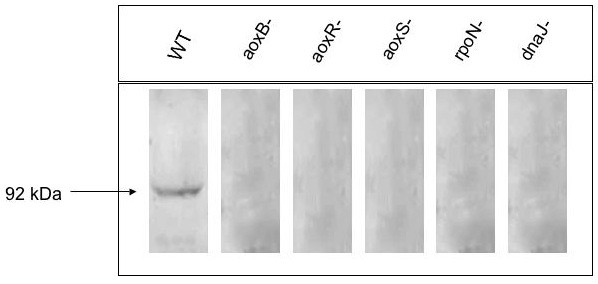
**Immunodetection of AoxB protein in total protein extracts of *H. arsenicoxydans *wild-type and mutant strains**.

To get further insight into the involvement of AoxR, AoxS, RpoN and DnaJ in arsenite oxidase activity, Western immunoblotting experiments were performed using antibodies raised against AoxB. The abundance of this protein was evaluated from total protein extracts of *H. arsenicoxydans *wild-type and mutant strains grown in the presence or not of As(III). AoxB was detected as a single band corresponding to a molecular mass of 92 kDa in As(III)-challenged *H. arsenicoxydans *strain (Figure 4). This single band was not observed in the various mutant strains. Furthermore, arsenite oxidase activity on native gel was only detected in As(III)-challenged wild type total extract (data not shown). Taken together these results suggest that the lack of activity in the mutant strains is due to the absence of AoxB protein, which may result from an effect of AoxR, AoxS, RpoN and DnaJ on *aoxAB *expression.

### Effect of AoxR, AoxS, RpoN and DnaJ on the control of arsenite oxidase operon expression

To determine the involvement of *aoxR*, *aoxS, dnaJ *and *rpoN *on *aoxAB *transcription, we performed quantitative RT-PCR experiments. For each strain, changes in *aoxB *transcript abundance were compared to two internal controls, i.e. the putative RNA methyltransferase gene and the peptide deformylase gene, in cultures challenged or not by As(III). The expression of *aoxB *mRNA was increased by a 9.4 fold factor after As(III) exposure in the *H. arsenicoxydans *wild-type strain. In contrast, *aoxB *expression was not increased in Ha482 *(aoxS)*, Ha483 *(aoxR)*, Ha3109 *(rpoN) *and Ha2646 *(dnaJ) *mutant strains, suggesting that the corresponding proteins play a crucial role in *aoxAB *operon expression (Table 2).

**Table 2:** *aoxB *relative expression in *H. arsenicoxydans *wild-type and mutant strains.

Strain	*aoxB *expression ratio +As(III)/-As(III)	Standard error
Wild type	**9.406**	0.630
Ha3109 (*rpoN*)	0.250	0.060
Ha483 (*aoxR*)	0.111	0.024
Ha482 (*aoxS*)	0.200	0.029
Ha2646 (*dnaJ*)	1.156	0.289

Expression ratios of *aoxB *in *H. arsenicoxydans *wild-type and mutant strains without As(III) versus an As(III) 8 hours induction (1.33 mM), as measured by quantitative RT-PCR. Expression of each gene was normalized to the expression of the two housekeeping genes HEAR0118 and HEAR2922 coding for a peptide deformylase and a putative RNA methyltransferase, respectively. Standard errors were calculated from the data of two quantitative PCR replicates obtained from two independent biological replicates. Bold text indicates statistically significant induction.

### Molecular mechanisms of arsenite oxidase transcription

**Figure 5:**
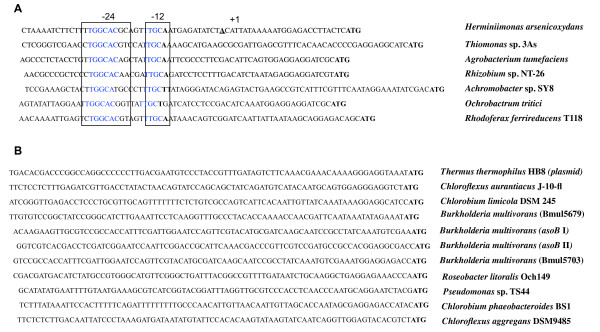
**Determination of *aoxA *transcription start site by 5'RACE and identification of a σ^54 ^consensus motif**. The transcription start site (TSS) of *aoxA *is in bold and indicated as +1 in the *aoxA *promoter sequence. The -12 and -24 boxes are highlighted and the consensus sequence is indicated in bold. The *aoxA *promoter was also aligned with the promoter sequences of *A. tumefaciens, Thiomonas *sp., *Rhizobium *sp. NT-26, *Achromobacter *sp., *R. ferrireducens, O. tritici*, *C. aurantiacus*, *C. limicola, T. thermophilus, B. multivorans, R. litoralis, Pseudomonas *sp.*TS44, C. phaeobacteroides *and *C. aggregans*. Two distincts sequences were shown A. DNA sequences with a σ^54^-dependent promoter motif (indicated in boxes). B. DNA sequences without a σ^54^-dependent promoter motif. Sequence informations of other genes were obtained from GenBank database and their localization on the chromosome or the plasmid is given by a nucleotide numbering. Their accession numbers are: *A. tumefaciens *(ABB51929.1)*, Thiomonas *sp. (ABY19317.1), *Rhizobium *sp. NT-26 (AAR05655.1), *Achromobacter *sp. (ABP63659.1), *R. ferrireducens *(YP_524326.1)*, O. tritici *(ACK38266.1), *C. aurantiacus *(YP_001634828.1), *C. limicola *(YP_001942455.1)*, T. thermophilus *(YP_145367.1)*, B. multivorans *(YP_001585660.1, YP_001941631.1, YP_001941634.1, YP_001585641.1)*, R. litoralis *(ZP_02142508.1)*, Pseudomonas *sp.*TS44 *(ACB05952.1)*, C. phaeobacteroides *(YP_001960746.1) and *C. aggregans *(YP_002461760.1).

The *aoxR *and *aoxS *genes encode a two-component system while *rpoN *encodes a sigma factor which recognizes a particular promoter with a specific -12/-24 binding site. These three proteins may therefore play a role in the initiation of *aoxAB *transcription. To get further insight into the molecular interactions between those regulators and the *aoxAB *promoter, we mapped the transcriptional start site of this operon by the amplification of *aoxAB *cDNA ends and 5'RACE. Messenger RNAs were extracted from induced (1.33 mM As(III)) and non induced *H. arsenicoxydans *wild-type strain cultures. A single transcriptional start site was identified from induced cells at -26 bp relative to the translation start codon, while no transcriptional start site was identified from non induced cells. In agreement with this, a T**GGCACG**CAGT**TTGC **putative -12/-24 σ^54^-dependent promoter motif was identified upstream of the *aoxAB *transcriptional start site (Figure 5). In addition, multiple alignment of *aoxAB *promoter sequences present in databases revealed a similarity to promoters recognized by σ^54 ^in *A. tumefaciens, Thiomonas *sp., *Rhizobium *sp. NT-26, *Achromobacter *sp., *Rhodoferax ferrireducens, Ochrobactrum tritici *(Figure 5A). In contrast, no such σ^54^-dependent promoter motif was found in several strains containing the *aoxAB *operon but lacking the two-component transduction system *aoxRS *operon, such as *Chloroflexus aurantiacus*, *Chlorobium limicola, Thermus thermophilus, Burkholderia multivorans, Roseobacter litoralis, Pseudomonas *sp.TS44*, Chlorobium phaeobacteroides *and *Chloroflexus aggregans *(Figure 5B).

**Figure 6:**
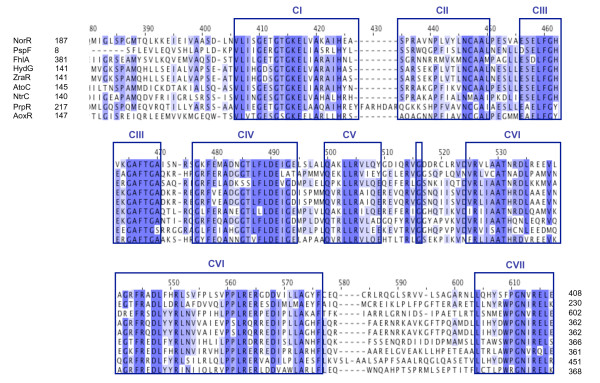
**Amino acids conservation between σ^54 ^Enhancer Binding Proteins (EBP) and AoxR**. Sequence alignment was performed with ClustalW. The conserved amino acids are presented with a blue background and the blue intensity reflects sequence similarities. Only the central binding domain is indicated. Region CI has remarkable similarity to the consensus glycine-rich flexible loop motif (Walker A - consensus motif GxxGxGK), and also contains hydrophobic residues. Region CII is hydrophobic. The region CIII is predicted to fold into two alpha helices separated by a turn. This region is involved in a specific interaction between the EBP and the Eσ^54 ^required for open promoter complex formation via the GAFTGA motif. Region CIV is rich in glycine, negatively charged and contains a consensus sequence of 4 aliphatic residues followed by 2 negatively charged residues (Walker B - consensus motif TVFLDE); in contrast, CVI is positively charged and is rich in aromatic residues and proline. Region CV is found about 80 amino acids away from region CI, and has a consensus sequence QakLLRVLqe. Finally, region CVII has a core of eight highly conserved amino acids. Sequence informations of other genes were obtained from Colibri database (Institut Pasteur, Paris).

Remarkably, a multiple alignment of amino acid sequences revealed that AoxR shares significant homology with a number of σ^54 ^RNA polymerase transcriptional activators, i.e. 35.96% identity with ZraR and 35.26% identity with AtoC from *E. coli *K12. AoxR contains three conserved domains shared by most Enhancer Binding Proteins (EBP), namely a N-terminal response regulator receiver domain (amino acids 18-130), a central σ^54 ^interaction domain (amino acids 147-368) common to all σ^54 ^dependent EBPs (Pfam E-value 10^-116^; http://www.sanger.ac.uk/cgi-bin/Pfam/getacc?PF00158) and a C-terminal DNA binding helix-turn-helix (HTF) domain (amino acids 421-463) enable to bind to specific upstream activation sequences [20]. AoxR shares similarities with several EBPs of σ^54 ^essential for the formation of an open complex formation during σ^54^-dependent transcriptional initiation, in particular the σ^54 ^activator sequence GAFTGA loop 1 which directly binds to σ^54 ^conserved region III (Figure 6) [21]. Taken together, these observations strongly suggest that AoxR interacts directly with RpoN to initiate the transcription of *aoxAB *operon in *H. arsenicoxydans*.

## Discussion

**Figure 7:**
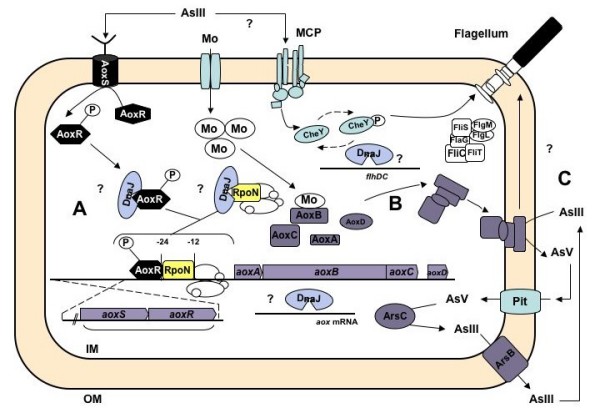
**Conceptual representation of the complex arsenite oxidation process in *H. arsenicoxydans***. Several major control mechanisms are involved: A. a transcriptional regulation: AoxS acts as a sensor of As(III) environmental signal and then phosphorylates AoxR. The phosphorylated AoxR binds to RpoN, which interacts with RNA polymerase. The RpoN-RNA polymerase complex with its AoxR co-activator initiates the *aox *operon transcription. DnaJ may regulate aox mRNA stability or act on the folding of AoxR or σ^54^; B. Then, arsenite oxidase is synthesized and exported by the TAT secretion system; C. consequently, arsenite oxidase exerts a key role in arsenic detoxification, by the transformation of the more toxic form As(III) into a less toxic form As(V). This process is known to affect motility, which may involve a MCP chemotaxis protein and requires the DnaJ co-chaperone. IM= Inner Membrane, OM= Outer membrane.

Despite many works devoted to arsenic metabolism in microorganisms, little is known about the regulation of arsenite oxidase activity. In the present study, the combination of transcriptomic, genetic and molecular data provided a comprehensive view of the role of various proteins in the control of arsenite oxidation in *H. arsenicoxydans *(Figure 7). We showed that some proteins play an indirect role in this process, as their presence is not essential for AoxAB synthesis. In this respect, the AoxB large subunit contains a Mo site required in arsenite oxidase enzymatic activity [22]. Ha3437 (*modC*) and Ha3438 (*modB*) mutations were located in the molybdenum high-affinity transport system operon, which further support the key role of this element in enzyme activity. In addition, the recovery of As(III) oxidase activity in these two mutants in the presence of an excess molybdenum suggests that Mo may also be transported through an alternative uptake system in *mod *mutants, e.g. a low-affinity uptake system involving non specific permeases such as HEAR0069, HEAR0154, HEAR1749 or HEAR2391 or a sulfate transport system, as described in *E. coli mod *mutants [23].

More importantly, our results suggest that AoxR and RpoN constitute a transcriptional complex that play a major role in the initiation of *aoxAB *operon transcription. Three mutants, i.e. Ha482 (*aoxS*), Ha483 (*aoxR*) and Ha3109 (*rpoN*), were affected in this process. The amino acids sequence analysis of *H. arsenicoxydans *AoxR and AoxS revealed the existence in these proteins of structural features common to partners of two-component signal transduction systems, which are composed of a sensor kinase and a response regulator [24]. Moreover, the comparison of AoxS and AoxR protein sequences with those of *A. tumefaciens *revealed similarities. Indeed, the AoxS protein sequence contains short blocks of conserved motifs that are consistent with a role of sensor histidine kinase, e.g. the "H" (amino acids 279 to 287: LAHEVNNPL), the "G2" (amino acids 435 to 441: GRIGLGL) and the "N" (amino acids 380 to 391: VRQIVLNLVLNA) domains. In addition, four highly invariant residues playing a central role in phosphorylation correspond to Asp9, Asp10, Asp57 and Lys107 in the *H. arsenicoxydans *AoxR protein. The three asparagine residues are known to interact together to form an "acid pocket", characteristic of orthodox receivers, into which the side chain of Lys109 protrudes [24]. Finally, a transmembrane region and a 17 amino acids residue cluster possibly exposed to the periplasm are present in AoxS and could serve as a signal receptor in the presence of As(III) in the medium. The detection of As(III) would then lead to AoxS autophosphorylation at a histidine residue via ATP hydrolysis and phosphotransfer to an aspartate residue in the response regulator AoxR, as recently proposed in *A. tumefaciens *[14].

Remarkably, our results demonstrated for the first time that the alternative N sigma factor (σ^54^) is essential for the initiation of arsenite oxidase transcription. Indeed, a mutation in the corresponding gene led to a complete loss of As(III) oxidation and *aoxB *transcription in Ha3109 (*rpoN*). σ^54 ^is one of the alternative sigma subunits of RNA polymerase responsible for specific binding to DNA. The core RNA polymerase complexed with σ^54 ^is usually associated with nitrogen assimilation and fixation, but is also known to play a role in various physiological processes, e.g. flagellar synthesis, carbon source utilization or bacterial virulence [25]. To date, only one report has shown that σ^54 ^participates in the transcription of genes possibly involved in metal tolerance, i.e. the *zraR/S *genes that code for a zinc and lead responsive two-component regulatory system in *E. coli *[26]. RNA polymerase together with σ^54 ^binds to a specific promoter site, with the consensus DNA sequence YTGGCACGNNNNTTGCWNNw [27], forming a transcriptionally inactive closed complex. Such a characteristic -12/-24 σ^54^-dependent promoter motif, i.e. T**GGCACG**CAGT**TTGC**, was identified 26 pb upstream of the transcriptional initiation codon of *aoxAB *with respect to the +1 transcriptional start site (Figure 5), which confirmed the need for RpoN in the initiation of *aoxAB *transcription. Changes in the conformation of σ^54^-RNA polymerase are nucleotide dependent. Indeed, the DNA melting step absolutely requires the interaction with a transcriptional activator protein. Most of these σ^54^-dependent activators share three domains found in AoxR, i.e. a C-terminal DNA binding domain that binds to upstream activation sequences, a conserved central domain belonging to the AAA+ (ATPases associated with various cellular activities) protein family to proceed with initiation of transcription and a N-terminal receiver domain that regulates its own AAA+ domain [20,28,29]. A multiple alignment of the central domain revealed a conservation of a common architecture between AoxR and σ^54 ^EBPs. Indeed, seven highly conserved sequence motifs corresponding to a σ^54 ^interaction domain of AoxR further support the direct interaction of AoxR with RpoN to stimulate the transcription of *aoxAB *operon in *H. arsenicoxydans *(Figure 6). This central σ^54 ^interaction domain has been already used to identify new σ^54 ^EBPs [30-37]. It contains the highly conserved signature sequence GAFTGA loop (conserved region CIII, Figure 6) proved to be required for direct interaction with σ^54 ^[21,38,39], the Walker A (conserved region I, Figure 6) and B (conserved region CIV, Figure 6) ATPase motifs necessary for the open complex formation.

Both multiple sequences alignment and genetic environment analysis of *aox *promoters suggest the existence of a wide diversity in the transcriptional control of the *aox *operon. Indeed, as in *H. arsenicoxydans*, a σ^54^-dependent promoter signature was identified in bacteria possessing a two-component transduction system AoxRS operon downstream of the *aoxAB *operon, e.g. *A. tumefaciens *and *O. tritici *(Figure 5A). In contrast, no σ^54^-dependent promoter motif and no *aoxR *homologous gene were found in other bacteria, e.g. *C. aurantiacus *or *C. aggregans *(Figure 5B). These observations suggest that the transcription of the *aox *operon in these bacteria may involve other regulatory proteins and that AoxR may represent a specific co-activator of RpoN in the initiation of the *aox *operon transcription. Finally, our results provide evidence that the DnaJ co-chaperone is required for As(III) oxidation. DnaJ is part of the DnaK-DnaJ-GrpE Hsp70 machinery. Hsp70 chaperones represent one of the most potent defence cellular mechanism against environmental insults as DnaK-DnaJ-GrpE are known to assist protein folding [40,41] or to be involved in mRNA stability [42]. In the present study we showed that there is no induction of *aoxAB *transcription in the *dnaJ *mutant, resulting in a loss of AoxAB synthesis.

Several possible mechanisms involving DnaJ in the regulation of arsenite oxidase can be hypothesized. DnaJ may be required for the proper folding or activity of the AoxR regulator. Such a function has been demonstrated for the positive regulator CRP in a *dnaJ *deletion mutant in *E. coli *[43]. Similarly, a post-transcriptional regulation of the arsenite oxidase itself can not be excluded. Moreover, a Tat (Twin-Arginine Translocation) signal has been detected in the AoxA sequence of *H. arsenicoxydans *[6]. Proteins secreted to the periplasm via a Tat protein export pathway are known to require a folding by Hsp70 chaperones before their secretion. DnaJ could be one of these chaperones [44,45]. Another possible target of DnaJ may be the RpoN sigma factor, as this chaperone has been demonstrated to play a role in the regulation of σ^S ^in various species [46]. Alternatively, several mechanisms are known to be involved in the stability of messenger RNA. For example, in *E. coli*, a long 5' untranslated region (UTR) has been observed upstream of the transcriptional start site of the *flhDC *flagellum master operon. This region plays a crucial role in the stability of the mRNA controlled by CsrA [19]. In the present report, the *aoxAB *transcriptional start site was located 26 bp upstream of the translational start codon, providing evidence that such a long 5'UTR does not exist upstream of the *aox *operon. However, this does not rule out a role of DnaJ on the stability of *aoxAB *mRNA, as HSP proteins are known to function as mRNA stabilizers and to protect them against nuclease degradation [42]. Further experiments will therefore be required to fully elucidate the molecular mechanisms of arsenite oxidase regulation in *H. arsenicoxydans*.

## Conclusion

Taken together, our observations provide evidence that multiple proteins play a role in the control of arsenite oxidation in *H. arsenicoxydans*. The following regulatory model is proposed: AoxS responds to the presence of As(III) in the environment and autophosphorylates. The phosphate is then transferred to AoxR, which acts as a positive regulator of the *aox *operon and activates the initiation of the transcription in association with RpoN. In addition, DnaJ acts on the expression or the stability of both arsenite oxidation and motility genes, demonstrating that these two functions are strongly linked. Our results include the role of RpoN and DnaJ in arsenite oxidase synthesis, which provide further insight into the molecular mechanisms used by *H. arsenicoxydans *to cope with the most toxic form of arsenic in its environment.

## Methods

### Bacterial strains and growth media

Bacterial strains used in this study are listed in Table 3. *H. arsenicoxydans *ULPAs1 was grown in a chemically defined medium (CDM), supplemented by 2% agar when required [4]. *Escherichia coli *S17-1 strain [47] was cultivated in LB medium (MP Biochemicals). Matings were performed on CDM to which 10% (wt/vol) LB medium was added, as previously described [9]. Tryptone swarm plates containing CDM supplemented with 1% Bacto-Tryptone and 0.25% agar were used to assess bacterial motility.

**Table 3:** Bacterial strains used in this study.

Name	Characteristics	Reference
***Escherichia coli***		
S17-1 (*-pyr*)	pUT/miniTn*5*::*lacZ2*	De Lorenzo *et al.*, 1990
***Herminiimonas arsenicoxydans***		
ULPAs1	Wild type	Weeger *et al.*, 1999
M1	*aoxA*::Tn*5lacZ2*	Muller *et al.*, 2003
M2	*aoxB*::Tn*5lacZ2*	Muller *et al.*, 2003
Ha482	*aoxS*::Tn*5lacZ2*	This work
Ha483	*aoxR*::Tn*5lacZ2*	This work
Ha3437	*modC*::Tn*5lacZ2*	This work
Ha3438	*modB*::Tn*5lacZ2*	This work
Ha2646	*dnaJ*::Tn*5lacZ2*	This work
Ha3109	*rpoN*::Tn*5lacZ2*	This work

### Transposon mutagenesis

The mini-Tn*5*::*lacZ2 *transposon [47] was delivered by mobilization of the suicide vector pUT/mini-Tn5::*lacZ2 *from *E. coli *S17-1 (*λ-pyr*) to *H. arsenicoxydans*. Conjugation was performed and transformants were selected as previously described [9].

### Selection of arsenite oxidase mutants

Mutants were screened for arsenite oxidase activity as previously described [9]. Agar plates were flooded with a 0.1 M AgNO_3 _solution to visualize arsenite oxidation [16]. Mutants affected in molybdenum metabolism were also tested on CDM agar plates supplemented with 50 μM Na_2_MoO_4_, 2H_2_O and 1.33 mM As(III).

### DNA manipulation and insertion mapping

DNA manipulations were carried out according to standard protocols, as described by Sambrook *et al*. [48]. Total DNA was isolated from mutant strains with the Wizard Genomic DNA purification kit (Promega). Transposon insertion sites were mapped as previously described [9]. Briefly, total DNA was digested and self-ligated. This ligation mixture was used as a template for PCR amplification using mini-transposon specific primers. The PCR products obtained were purified with a PCR purification kit (Qiagen) and sequenced on an Applied Biosystems ABI prism 3130×l capillary sequencer. The resulting sequences were compared to the *H. arsenicoxydans *genome sequence [6] to identify disrupted CDS. Finally, insertion sites and transposon orientations were precisely mapped by sequencing PCR products obtained with two primers hybridizing upstream and downstream, respectively, of the insertion site of each disrupted gene (see Additional file 2, Table S2).

### Arsenic speciation determination

*H. arsenicoxydans *wild type and mutants were grown for 48 hours in CDM medium supplemented with 1.33 mM As(III). Culture supernatants were filtered through sterile 0,22 μm pore size filters (VWR). Arsenic species were separated by high-performance liquid chromatography (HPLC) and quantified by inductively coupled plasma-atomic emission spectrometry (ICP-AES), as previously described [9].

### RNA extraction

Strains were grown at 25°C for 24 h (OD = 0,15) and cultures were induced by addition of 0.66 mM or 1.33 mM As(III) for 8 hours before extraction. Samples were harvested and stored at -80°C. RNA was extracted as previously described [7]. After extraction procedure, RNA integrity was checked by electrophoregram analysis on a BioAnalyser (Agilent) and total RNA concentration was determined spectrophotometrically with a Nanodrop.

### Microarrays and data analysis

Microarrays containing 60-mer oligonucleotides for all predicted *H. arsenicoxydans *genes http://www.genoscope.cns.fr/agc/mage/arsenoscope were used, as previously described [7]. Briefly, total RNA (5 μg) was reverse transcribed and indirectly labelled according to manufacturer's instructions with some modifications [7]. The quality and concentration determination as well as hybridization and scanning were performed as previously described [7] Three distinct biological RNA samples were prepared from in each growth condition (with and without As(III) induction) and labelled either by Cy3 or Cy5 in a dye-swap design. Microarray data were deposited in ArrayExpress (accession E-MEXP-2199 and A-MEXP-1594). Data normalization and statistical analysis were performed as previously described [7]. Briefly, data were acquired and analyzed by Genepix Pro 6.0 (Axon Instrument). The experiment design included three biological replicates. For each of them, induced and non-induced cells were compared in dye swap experiments. The resulting arrays were analyzed using the R software http://www.r-project.org. A slide by slide Loess normalization was performed using the limma package [49]. Valid log2 expression ratios from replicated spots were averaged on each array so as to get statistically independent ratios for each oligonucleotide included in the array design. For the same reason, dye swap arrays were also averaged. Oligos that had no valid expression ratios on the ten arrays were excluded from the data set for further analysis, which was carried out using the varmixt package and the VM option [50]. The resulting raw *p*-values were adjusted according to a Benjamini and Yekutieli procedure [51]. Genes showing a valid *p*-value and a more than two-fold decreased or increased expression were considered as differentially expressed between the two conditions and were retained for further study.

### Quantitative real time PCR

Quantitative PCR experiments were performed with RNA prepared as described for microarrays. RNA aliquots were purified with the RNeasy Plus mini kit (Qiagen) to ensure the elimination of genomic DNA. Total RNA concentration was determined spectrophotometrically using a Nanodrop and RNA integrity was electrophoretically verified. Total RNA (1,9 μg) was reverse transcribed with SuperScript III first-strand synthesis system for RT-PCR (Invitrogen) using random hexamers. Real time quantitative PCR was carried out with a MyiQ single-color Real-time PCR detection system. The reaction mixture contained 12,5 μl of MESA Blue qPCR MasterMix Plus for SYBR Assay with fluorescein (Eurogentec), 5 μl of cDNA and 300 nM of each primer in a total volume of 25 μl. Thermocycling conditions were as follow: 5 min at 95°c and 40 cycles of 15 s at 95°C, 15 s at 61°c and 1 min at 72°C. The PCR efficiency of the genes of interest and internal control genes were optimized to be similar enough by adjusting the primer concentrations to 300 nM each (data not shown). For each quantitative PCR run, non-template controls were performed to identify false positives and negative controls without reverse transcriptase were performed for each cDNA synthesis reaction and verified in real time PCR to determine the presence of contaminating genomic DNA. Two biological replicates (independent cultures) and two quantitative PCR replicates were performed for each experience. Amplification products were designed to be less than 175 bp in size. The pairs of primers used are listed in Additional file 2, Table S2. Two housekeeping genes, i.e. HEAR2922 coding for a putative RNA methyltransferase and HEAR0118 coding for a peptide deformylase, were used as standards to obtain normalized *aoxB *(HEAR0478) gene ratio [52] in the As(III) induced sample compared to the non-induced sample. These two housekeeping genes showed a stable expression between the two analyzed conditions (without As(III) and after an 8 hours As(III) exposure) when observing the microarrays data. The data were analyzed with the Relative Expression Software Tool [53]. Statistical significance was defined as a *p*-value of ≤ 0.05.

### 5'RACE experiment

The transcriptional start site of *aoxAB *operon was determined using the 5'RACE system for rapid amplification of cDNA ends (Invitrogen). Total RNA was obtained as described before. Reverse transcriptase reactions were performed using 5 μg total RNA and a gene specific reverse primer (5'-CATGGGCACTTGAATGTCTTG-3'). Reactions were heated at 70°C for 10 min and immediately prewarmed at 50°C before addition of Super-Script II reverse transcriptase. Reverse transcription was conducted at 50°C for 50 min and stopped at 70°c for 15 min. Purification and tailing of cDNA were performed according to manufacturer's instructions. The resulting cDNA was amplified by PCR using the provided Abridged Anchor Primer and a gene specific primer (5'-ATGCTGTGCGCGACGATATCG-3') located upstream of the original cDNA primer.

### Preparation of protein extracts, SDS-PAGE and PAGE separation

Western immunoblotting were performed from late exponential phase wild-type and mutant strains grown in 1 liter CDM (with and without the presence of 100 mg/liter As(III)). The cultures were harvested by centrifugation for 10 min at 9,000 × *g*. Cell pellets were resuspended in distilled water and sonicated at 100 A (15 times 1 min with 1 min interval on ice, 80% duty cycle). Cell debris were removed by centrifugation (15 min at 13,000 × *g*). The supernatant was collected (total extract) and stored at -20°C. The protein concentration of each sample was measured with a Bio-Rad protein assay kit. First, fifty micrograms of each protein extract was loaded onto an 11% polyacrylamide-SDS gel. Second, fifty micrograms of each protein extract were loaded onto a polyacrylamide gel (native gel). The assay of arsenite oxidase activity followed the transfer of reducing equivalents from arsenite to 2,4-dichlorophenolindophenol (DCIP) as described by Anderson *et al*. [54]. Briefly, the reduction of DCIP (60 μM) was monitored in the presence of 200 μM sodium arsenite in 50 μM MES, pH 6.0, at 25°C.

### Preparation of antibodies and Western blot analysis

Monoclonal antibodies raised against an AoxB peptide were obtained from Proteogenix. Briefly, a hexadecapeptide with the SKNRDRVALPPVNAQK sequence was synthesized. This peptide corresponds to the N-terminal 16 amino acids of the arsenite oxidase large subunit of *H. arsenicoxydans*. The peptide was then coupled to keyhole limpet haemocyanin (KLH). Two rabbits were injected at multiple subcutaneaous sites with peptide-KLH at 14 days intervals. Animals were prebled at day 0, bled at day 49 (from an ear vein) and totally bled at day 90. Antibodies were partially purified on an affinity column substituted with the peptide.

After SDS-PAGE electrophoresis, the proteins were electrotransfered to a nitrocellulose membrane (Schleicher and Schuell, BA-85) using a Trans-Blot system (Bio-Rad) at 100 V, 4°C for 1 h. The membranes were washed twice in Tris buffered saline (TBS: 10 mM Tris-HCl pH7.5, 150 mM NaCl) and blocked in TBS with 0,3% bovine serum albumin (BSA). The membrane was then washed three times in Tris-buffered saline with TritonX100 and Tween 20 (TBS-T: 20 mM TrisHCl pH7.5, 500 mM NaCl, 0,2% Triton X-100, 0,05% Tween20), and incubated for 1 h with the AoxB antisera (1:800 dilution) in TBS-T with 0,3% BSA. Excess antibodies were removed by repeated washing with TBS-T. After 30 min incubation in TBS-T containing the secondary antibody (1:800 dilution of goat IgG against rabbit IgG, Sigma) conjugated with alkaline phosphatase, the membrane was washed twice with TBS-T and revealed by NBT/BCIP color reagent using standard procedures.

## Authors' contributions

SK and JCA wrote the manuscript and performed the genetic experiments. SK carried out the quantitative PCR and 5' RACE experiments. JCA performed the Western immunoblotting analysis. CP, OS, MAD and JYC conceived and performed the transcriptomic experiments and the data analyses. DL performed the chemical experiments. FGC, FH, FAP and PB helped to analyze the data and critically revised the manuscript. PB coordinated and conceived the study. All authors read and approved the final manuscript.

## Supplementary Material

Additional file 1 **Supplemental table S1**. Selected genes differentially expressed after 8 hours arsenite stress. Click here for file

Additional file 2 **Supplemental table S2**. Oligonucleotides used in the study. A. Identification of transposon insertion sites in *H. arsenicoxydans *mutants. B. Quantitative RT-PCR. Click here for file
