# Erratum to: Multiple controls affect arsenite oxidase gene expression in Herminiimonas arsenicoxydans

**DOI:** 10.1186/s12866-017-0976-8

**Published:** 2017-03-28

**Authors:** Sandrine Koechler, Jessica Cleiss-Arnold, Caroline Proux, Odile Sismeiro, Marie-Agnès Dillies, Florence Goulhen-Chollet, Florence Hommais, Didier Lièvremont, Florence Arsène-Ploetze, Jean-Yves Coppé, Philippe N Bertin

**Affiliations:** 1 UMR7156 Génétique Moléculaire, Génomique et Microbiologie, CNRS Université de Strasbourg, 28 rue Goethe, 67000 Strasbourg, France; 2 Plate- forme technologique Puces à ADN, Institut Pasteur, 28 rue du Dr. Roux, 75724 Paris cedex 15, France; 3 UMR5240 Microbiologie, Adaptation et Pathogénie, CNRS Université Lyon 1, Bâtiment André Lwoff, 10 rue Dubois, 69622 Villeurbanne cedex, France

## Erratum

After the publication of our article [1], similarities between lanes 2, 5 and 6 (aoxB-, rpoN- and dnaJ-) and between lanes 3 and 4 (aoxR- and aoxS-) in Fig. 4 were brought to our attention. This error occurred during the compilation of the Western blot images. A corrected version of Fig. 4 has been assembled from the original Western blot experiments and is presented below. This error affects neither the other results nor the conclusions of the article.

**Fig. 4:**
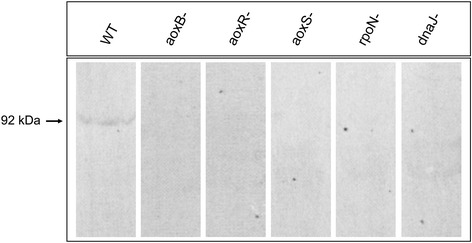
Immunodetection of AoxB protein in total protein extracts of *H. arsenicoxydans* wild-type and mutant strains
