# Supplementary material for: Multiple controls affect arsenite oxidase gene expression in Herminiimonas arsenicoxydans
Source: BMC Microbiol. 2010 Feb 18;10:53. doi: 10.1186/1471-2180-10-53 (PMC2848651; doi:10.1186/1471-2180-10-53)
Supplement: Additional file 1 — Supplemental table S1. Selected genes differentially expressed after 8 hours arsenite stress. [file 1471-2180-10-53-S1.PDF]

Additional material

Supplemental table S1. Selected genes differentially expressed after 8 hours arsenite stress. (%) represents the part of genes up- or down-regulated in any functional category.

A- UP-REGULATED

GENE NUMBER		PROTEIN FUNCTION	FOLD CHANGE
**UNKNOWN FUNCTION (30,7%)**			
HEAR0027		Hypothetical protein	2,1
HEAR0055		Hypothetical protein	2,2
HEAR0160		Putative exported alkaline phosphatase	13,7
HEAR0190		Hypothetical protein	2,3
HEAR0474		Conserved hypothetical protein	2,4
HEAR0484		Hypothetical protein	15,2
HEAR0487		Conserved hypothetical protein; putative exported protein	128,7
HEAR0493		Conserved hypothetical protein; putative GGDEF domain	22,3
HEAR0494		Conserved hypothetical protein	26,6
HEAR0495		Conserved hypothetical protein	17,7
HEAR0497		Hypothetical protein; putative exported protein	31,9
HEAR0582		Conserved hypothetical protein; putative RNA-binding protein	2,9
HEAR0583		Conserved hypothetical protein	2,5
HEAR0584		Conserved hypothetical protein; putative exported protein	2,1
HEAR0613		Conserved hypothetical protein, putative membrane protein	10,8
HEAR0655		Conserved hypothetical protein; putative EAL and GGDEF domains	4,8
HEAR0923		Hypothetical protein	2,3
HEAR1023		Conserved hypothetical protein; putative EAL, GGDEF, CBS domains	13,8
HEAR1024		Hypothetical protein	6,9
HEAR1026		Conserved hypothetical protein; putative cytochrome c domain	3,4
HEAR1027		Conserved hypothetical protein, putative sulfite oxidase	3,6
HEAR1066		Conserved hypothetical protein	3,2
HEAR1097		Putative DedA protein	4,9
HEAR1100		Conserved hypothetical protein	69,8
HEAR1101		Putative Metallo-dependent phosphatase	15,4
HEAR1104		Conserved hypothetical protein	4,4
HEAR1114		Hypothetical protein	3,0
HEAR1292		Hypothetical protein	2,0
HEAR1376		Conserved hypothetical protein	2,4
HEAR1414		Conserved hypothetical protein ; putative exported protein	2,7
HEAR1416		Conserved hypothetical protein	2,4
HEAR1483		Conserved hypothetical protein, putative exported protein	2,1
HEAR1730		Conserved hypothetical protein	2,4
HEAR1731		Conserved hypothetical protein	4,0
HEAR1871		Conserved hypothetical protein	2,2
HEAR1967		Conserved hypothetical protein	3,1
HEAR2183		Conserved hypothetical protein; putative exported protein	2,1
HEAR3206		Conserved hypothetical protein	3,8
HEAR3427		Hypothetical protein	2,0
HEAR3463		Hypothetical protein	2,8
HEAR3464		Hypothetical protein	3,6
**PROTEIN FATE (0,8%)**			
HEAR0579		Putative protein-disulfide isomerase	2,2
**REGULATORY FUNCTION (2,3%)**			
HEAR0492	*phoB1*	Phosphate regulon transcriptional regulatory protein PhoB	38,1
HEAR0496		Putative serine phosphatase	25,9
HEAR0783		Putative transcription regulator aminotransferase GntR-related	2,0

CELLULAR PROCESSES (26,3%)

HEAR0191		Putative phosphate starvation-inducible protein *psiF* precursor	4,7
HEAR0476	*aoxD*	Cytochrome c-552 precursor (Cytochrome c552)	9,3
HEAR0477	*aoxC*	Putative nitroreductase	11,3
HEAR0478	*aoxB*	Arsenite oxidase large subunit (AOI)	32,0<br>* (9,4)
HEAR0479	*aoxA*	Arsenite oxidase small subunit precursor , Rieske type subunit, twin arginine translocation peptide	54,9
HEAR0482	*aoxS*	Signal transduction protein involved in AoxAB regulation (AoxS)	3,1<br>* (1,9)
HEAR0483	*aoxR*	AoxR regulatory protein	3,3<br>* (2,2)
HEAR0499	*arsR*	Arsenical resistance transcriptional regulator	40,2
HEAR0500	*arsC*	Arsenate reductase	50,1
HEAR0501	*arsB*	Arsenical pump (Ars family)	19
HEAR0502	*arsC*	Arsenate reductase	27,8
HEAR0503		Putative NADPH-dependent FMN reductase, ArsH-like	20,4
HEAR0538	*copR*	Transcriptional regulatory protein CusR	2,1
HEAR0581		Putative methyl-accepting chemotaxis protein	22,0
HEAR1428		Putative transcriptional regulator (ArsR family)	3,4
HEAR1620	*osmC*	Hydroperoxide resistance protein OsmC	2,2
HEAR1866	*fliC*	Flagellin	5,0
HEAR1867		Putative flagellar protein FlaG	4,0
HEAR1869	*fliS*	Flagellar protein	2,5
HEAR1870		Putative flagellar protein FliT	2,4
HEAR1890		Putative flagellar hook-associated protein 3 FlgL-like	2,2
HEAR1903		Putative negative regulator of flagellin synthesis (Anti-sigma-28 factor) FlgM-like	2,1
HEAR1959	*arsH*	NADPH-dependent FMN reductase, ArsH-like	4,9
HEAR1960	*arsCb*	Arsenate reductase	6,9
HEAR1962	*acr3*	Arsenite efflux pump ACR3	2,3
HEAR1963	*arsCa*	Arsenate reductase	22,8
HEAR1965	*arsR*	Arsenical resistance transcriptional regulator	2,6
HEAR2711	*acrR*	Putative transcriptional regulator *acrR* (Potential *acrAB* operon repressor)	3,3
HEAR3207	*arsC*	Arsenate reductase	44,0
HEAR3208		Putative *arsH*	30,5
HEAR3300	*arsH*	NADPH-dependent FMN reductase, ArsH-like	12,2
HEAR3301	*arsB*	Arsenical pump membrane protein	5,2
HEAR3302	*arsC*	Arsenate reductase	34,4
HEAR3303	*arsR1*	Arsenical resistance transcriptional regulator	21,5
HEAR3392	*ibp*	Small heat shock protein Hsp20	3,1

* Expression ratios of theses genes in *H. arsenicoxydans* wild type strain without As(III) versus an 1.33mM As(III) 8h induction measured by quantitative RT-PCR. Results are indicated in bold. Data were analyzed with the Relative Expression Software Tool using the two housekeeping genes HEAR0118 and HEAR2922.

TRANSPORT AND BINDING PROTEIN (26,3%)

HEAR0104		Putative TRAP-type C4-dicarboxylate transport system, large permease component	2,2
HEAR0159		Putative Permease of the major facilitator superfamily	3,5
HEAR0481		Putative phosphite transport system-binding protein PtxB precursor	3,7
HEAR0486		ABC-type phosphate transport system, periplasmic component	126,7
HEAR0488		Putative ABC-type phosphate transport system, permease component	84,9
HEAR0489		Putative ABC-type phosphate transport system, auxiliary and permease component	48,8
HEAR0490	*pstB2*	Phosphate import ATP-binding protein	60,6
HEAR0491		Putative phosphate uptake regulator PhoU	72,5
HEAR0498	*sphX*	Phosphate-binding periplasmic protein precursor (PBP)	49,2

HEAR0504		Putative amino-acid metabolite efflux pump	8,9
HEAR0505		Putative Na+-dependent transporter	2,3
HEAR0656		Putative ABC-type phosphonate transport system, periplasmic component	3,7
HEAR0784		Putative threonine efflux protein	4,4
HEAR0785		Putative permease of the drug/metabolite transporter (DMT)	4,3
HEAR1098		Putative ABC-type branched-chain amino acid transport systems, periplasmic component	14,6
HEAR1107	*pstS2*	Phosphate-binding periplasmic protein precursor (PBP)	16,6
HEAR1108	*pstC2*	Phosphate transport system permease protein PstC	10,8
HEAR1109	*pstA2*	Phosphate transport system permease protein PstA	12,0
HEAR1110	*pstB2*	Phosphate import ATP-binding protein PstB (Phosphate-transporting ATPase) (ABC phosphate transporter)	11,8
HEAR1111	*phoU2*	Phosphate transport system protein PhoU	12,6
HEAR1112	*phoB2*	Phosphate regulon transcriptional regulatory protein PhoB	9,7
HEAR1113	*phoR2*	Phosphate regulon sensor protein PhoR	4,3
HEAR1156		Putative ABC-type Fe3+ transport system	2,4
HEAR1389		Putative shikimate transporter	3,5
HEAR1466		Putative TRAP dicarboxylate transporter- DctP subunit	2,1
HEAR1501		Putative Fe2+ transport system protein A FeoA	2,2
HEAR1961		Putative permease of the major facilitator superfamily	5,0
HEAR2560		Putative Mg2+ and Co2+ transporter	4,1
HEAR3209		Putative sulfate transporter	2,4
HEAR3211	*ptxA*	Phosphonates import ATP-binding protein PhnC	3,8
HEAR3212	*ptxB*	Phosphonate transport system substrate-binding protein precursor	19,8
HEAR3213	*ptxC*	Putative phosphonates transport system permease protein phnE-like	14,7
HEAR3214	*ptxD*	Phosphonate dehydrogenase (NAD-dependent phosphite dehydrogenase)	14,9
HEAR3215	*ptxE*	Putative HTH-type transcriptional regulator protein PtxE-like	7,0
HEAR3299		Putative Heavy metal transport/detoxification protein	7,4
**METABOLISM (10,5%)**			
Energy metabolism (6%)			
HEAR0577	*phbA*	Acetyl-CoA acetyltransferase (Acetoacetyl-CoA thiolase)	2,1
HEAR1180	*iorB*	Isoquinoline 1-oxidoreductase, beta subunit	2,3
HEAR1193	*soxA*	Putative Cytochrome c SoxA	2,0
HEAR1375	*cydB*	Cytochrome d ubiquinol oxidase subunit 2 (Cytochrome d ubiquinol oxidase subunit II) (Cytochrome bd-I oxidase subunit II)	2,1
HEAR1412		Putative formate dehydrogenase subunit A	2,5
HEAR1413	*fdhB*	Formate dehydrogenase iron-sulfur subunit	2,6
HEAR1415		Putative formate dehydrogenase subunit C	2,9
HEAR2486	*hppA*	Pyrophosphate-energized proton pump (Pyrophosphate-energized inorganic pyrophosphatase) (H(+)-PPase) (Membrane-bound proton-translocating pyrophosphatase)	4,0
Central intermediary metabolism (3,7%)			
HEAR0270		Putative carboxymethylenebutenolidase	2,4
HEAR1102	*gtrA*	Glycosyltransferase	41,4
HEAR1103		Putative L-asparaginase	3,0
HEAR1106	*ppk*	Polyphosphate kinase (Polyphosphoric acid kinase) (ATP-polyphosphate phosphotransferase)	11,3
HEAR1199		Putative fumarylacetoacetate (FAA) hydrolase family	2,3
DNA metabolism (0,8%)			
HEAR0580		Putative DNA-polymerase/ribonuclease/exonuclease	2,6
**MOBILE AND EXTRACHROMOSOMAL ELEMENT FUNCTIONS (2,3%)**			
HEAR2113		Transposase IS21 family (partial)	2,7
HEAR3210		Transposase IS30 family	2,1
HEAR3216		Transposase IS30 family	2,1
**SIGNAL TRANSDUCTION (0,8%)**			

HEAR1105	Putative phosphohistidine phosphatase *sixA*	15,5

B- DOWN-REGULATED

GENE NUMBER		PROTEIN FUNCTION	FOLD CHANGE
**UNKNOWN FUNCTION (11,3%)**			
HEAR0831		Conserved hypothetical protein	-2,2
HEAR0870		Conserved hypothetical protein	-2,5
HEAR1099		Conserved hypothetical protein	-2,8
HEAR1456		Hypothetical protein	-2,6
HEAR1505		Hypothetical protein	-2
HEAR1663		Conserved hypothetical protein	-2,7
HEAR1812		Conserved hypothetical protein, putative membrane protein	-3,8
HEAR2080		Conserved hypothetical protein ; putative metal-binding protein	-2,1
HEAR2111		Conserved hypothetical protein, putative acyltransferase	-3,1
HEAR2112		Conserved hypothetical protein	-2,6
HEAR2135		Conserved hypothetical protein	-2,1
HEAR2393		Conserved hypothetical protein	-2,7
HEAR2483		Conserved hypothetical protein, putative permease	-2,3
HEAR2494		Hypothetical protein	-2,6
HEAR2495		Putative ATPase of the AAA+ class	-2,2
HEAR2949		Conserved hypothetical protein ; putative membrane protein	-2,3
HEAR3348		Conserved hypothetical protein	-2
HEAR3470		Conserved hypothetical protein	-2,2
**PROTEIN SYNTHESIS (41,3%)**			
HEAR0240	*rplM*	50S ribosomal protein L13	-4,2
HEAR0241	*rpsI*	30S ribosomal protein S9	-5,3
HEAR0439	*rpsU*	30S ribosomal subunit protein S21	-3,6
HEAR0624	*rimM*	16S rRNA processing protein	-2
HEAR0625	*trmD*	tRNA (guanine-N(1)-)-methyltransferase) (M1G-methyltransferase) (tRNA [GM37] methyltransferase)	-2
HEAR0626	*rplS*	50S ribosomal subunit protein L19	-6,7
HEAR0703	*rpmE2*	50S ribosomal protein L31 type B	-4,8
HEAR0845	*ileS*	Isoleucine tRNA synthetase (Isoleucine—tRNA ligase) (IleRS)	-2,4
HEAR1335	*rpsB*	30S ribosomal subunit protein S2	-6,7
HEAR1336	*tsf*	Elongation factor Ts (EF-Ts)	-3,7
HEAR1796	*pheT*	Phenylalanyl-tRNA synthetase beta chain (Phenylalanine—tRNA ligase beta chain) (PheRS)	-2,8
HEAR1797	*pheS*	Phenylalanyl-tRNA synthetase alpha chain (Phenylalanine—tRNA ligase alpha chain) (PheRS)	-2,6
HEAR1798	*rplT*	50S ribosomal subunit protein L20	-3,8
HEAR1799	*rpmI*	50S ribosomal protein L35	-2,2
HEAR1830	*rpsO*	30S ribosomal subunit protein S15	-2,4
HEAR2159	*rplI*	50S ribosomal subunit protein L9	-4,8
HEAR2160	*rpsR*	30S ribosomal protein S18	-7,7
HEAR2162	*rpsF*	30S ribosomal subunit protein S6	-3,3
HEAR2230	*lysS*	Lysyl-tRNA synthetase (Lysine—tRNA ligase) (LysRS)	-2,1
HEAR2328		Putative ATP-dependent RNA helicase	-2,8
HEAR2430	*typA*	GTP-binding protein TypA/BipA (Tyrosine phosphorylated protein A)	-2,3
HEAR2433	*infB*	Translation initiation factor IF-2	-2,3
HEAR2451	*rpsT*	30S ribosomal subunit protein S20	-2,6
HEAR2469	*rpmB*	50S ribosomal subunit protein L28	-2,9
HEAR2470	*rpmG*	50S ribosomal subunit protein L33	-3,1
HEAR2574	*rpsA*	30S ribosomal subunit protein S1	-6,7
HEAR2786	*rpmA*	50S ribosomal subunit protein L27	-2,9
HEAR2787	*rplU*	50S ribosomal subunit protein L21	-2,8
HEAR2894	*rplY*	50S ribosomal protein L25 (General stress protein CTC)	-6,7
HEAR3140	*rplQ*	50S ribosomal protein L17	-5,3

HEAR3142	*rpsD*	30S ribosomal subunit protein S4	-5,3
HEAR3143	*rpsK*	30S ribosomal subunit protein S11	-7,7
HEAR3144	*rpsM*	30S ribosomal subunit protein S13	-5,6
HEAR3145	*infA*	Translation initiation factor IF-1	-5,6
HEAR3147	*rplO*	50S ribosomal subunit protein L15	-8,3
HEAR3148	*rpmD*	50S ribosomal subunit protein L30	-10
HEAR3149	*rpsE*	30S ribosomal subunit protein S5	-11,1
HEAR3150	*rplR*	50S ribosomal subunit protein L18	-11,1
HEAR3151	*rplF*	50S ribosomal subunit protein L6	-9,1
HEAR3152	*rpsH*	30S ribosomal protein S8	-11,1
HEAR3153	*rpsN*	30S ribosomal subunit protein S14	-11,1
HEAR3154	*rplE*	50S ribosomal subunit protein L5	-7,7
HEAR3155	*rplX*	50S ribosomal subunit protein L24	-11,1
HEAR3156	*rplN*	50S ribosomal subunit protein L14	-11,1
HEAR3157	*rpsQ*	30S ribosomal subunit protein S17	-9,1
HEAR3158	*rpmC*	50S ribosomal protein L29	-11,1
HEAR3159	*rplP*	50S ribosomal subunit protein L16	-10
HEAR3160	*rpsC*	30S ribosomal protein S3	-10
HEAR3161	*rplV*	50S ribosomal subunit protein L22	-9,1
HEAR3162	*rpsS*	30S ribosomal subunit protein S19	-11,1
HEAR3163	*rplB*	50S ribosomal subunit protein L2	-9,1
HEAR3164	*rplW*	50S ribosomal subunit protein L23	-11,1
HEAR3165	*rplD*	50S ribosomal subunit protein L4	-10
HEAR3166	*rplC*	50S ribosomal subunit protein L3	-6,7
HEAR3167	*rpsJ*	30S ribosomal subunit protein S10	-6,7
HEAR3168	*tufB*	Elongation factor Tu-B (EF-Tu-B)	-4
HEAR3169	*fusA*	Elongation factor G (EF-G)	-4,8
HEAR3170	*rpsG*	30S ribosomal subunit protein S7	-5
HEAR3171	*rpsL*	30S ribosomal subunit protein S12	-5,3
HEAR3174	*rplL*	50S ribosomal subunit protein L7/L12	-7,1
HEAR3175	*rplJ*	50S ribosomal subunit protein L10	-11,1
HEAR3176	*rplA*	50S ribosomal subunit protein L1	-6,7
HEAR3177	*rplK*	50S ribosomal subunit protein L11	-7,1
HEAR3180	*tufB2*	Elongation factor Tu-B (EF-Tu-B)	-4
HEAR3471		Putative ribonuclease P rnpA	-2,5
HEAR3472	*rpmH*	50S ribosomal subunit protein L34	-2,2
**TRANSPORT AND BINDING PROTEIN (7,5%)**			
HEAR0305		Putative Preprotein translocase subunit YajC	-2,2
HEAR0387		Putative ABC type branched chain amino acid transport systems	-2,1
HEAR0388		Putative ABC transporter, ATPase component LivG	-2,2
HEAR0389		Putative ABC transporter, ATPase component LivG	-2,2
HEAR0390		Putative ABC-type branched-chain amino acid transport systems, periplasmic component	-3,6
HEAR0959		Putative TonB-like receptor precursor	-2
HEAR1916	*glcA*	L-lactate permease	-2,2
HEAR2485		Putative lactate permease	-2,1
HEAR2499	*cysP*	Thiosulfate-binding protein precursor	-2,6
HEAR3146	*secY*	Preprotein translocase SecY subunit	-4,5
HEAR3179	*secE*	Preprotein translocase subunit SecE	-3,4
HEAR3469	*oxaA*	Inner membrane protein OxaA	-3,2
**PURINES, PYRIMIDINES, NUCLEOSIDES AND NUCLEOTIDES (1,9%)**			
HEAR1260	*ndk*	Nucleoside diphosphate kinase	-2
HEAR1811		Putative ADP-ribose pyrophosphate	-2,2
HEAR2893	*prs*	Ribose-phosphate pyrophosphokinase (RPPK) (Phosphoribosyl pyrophosphate synthetase) (P-Rib-PP synthetase) (PRPP synthetase)	-2,3
**BIOSYNTHESIS OF COFACTORS, PROSTHETIC GROUPS, AND CARRIERS (1,2%)**			
HEAR1123		Putative bifunctional protein glutamate 1-semialdéhyde 2,1-aminomutase acylneuraminate cytidyllyltransferase	-2,2
HEAR2397	*cobA2*	Uroporphyrinogen-III C-methyltransferase	-2,3

**AMINO ACID BIOSYNTHESIS (6,2%)**			
HEAR0871	*argG*	Argininosuccinate synthetase (Citrulline—aspartate ligase)	-2,5
HEAR1035	*leuA1*	2-isopropylmalate synthase	-2,7
HEAR1165	*carB*	Carbamoyl phosphate	-2
HEAR2136	*metE*	5-methyltetrhydropteroyl triglutamate-homocystéine methyltransferase	-2,6
HEAR2150	*hom*	Homoserine dehydrogenase	-2,3
HEAR2577	*tyrA*	Prephenate dehydrogenase	-2
HEAR2578	*hisC2*	Histidinol phosphateaminotransferase	-3,1
HEAR2580	*serC*	Phosphoserine aminotransferase (Phosphohydroxythreonine aminotransferase) (PSAT)	-2,3
HEAR2948	*metF*	5,10-methylenetetrahydrofolate reductase	-2,3
HEAR2950	*sahH*	Adenosylhomocysteinase (S-adenosyl-L-homocysteine hydrolase) (AdoHcyase)	-4
**REGULATORY FUNCTION (3,8%)**			
HEAR0442		Putative RNA polymerase sigma factor, sigma70 factor *rpoD*	-2,2
HEAR0705	*rho*	Transcription termination factor (ATP-dependent helicase *rho*)	-2,8
HEAR1166	*greA*	Transcription elongation factor	-2,3
HEAR1457		Putative transcription elongation factor GreA	-3,2
HEAR3049		Putative stringent starvation protein A	-2,3
HEAR3072		Putative stress induced morphogen BolA protein	-2,1
**METABOLISM (21,8%)**			
Central intermediary metabolism (6,2%)			
HEAR0330	*pntB*	NAD(P) transhydrogenase subunit beta (Pyridine nucleotide transhydrogenase subunit beta) (Nicotinamide nucleotide transhydrogenase subunit beta)	-2,7
HEAR0343	*sucC*	Succinyl-CoA synthetase, beta subunit	-2,4
HEAR0344	*sucD*	Succinyl-CoA ligase [ADP-forming] subunit alpha (Succinyl-CoA synthetase subunit alpha) (SCS-alpha)	-2,7
HEAR0850	*gap*	Glyceraldehyde 3-phosphate dehydrogenase	-2,2
HEAR2380	*glyA*	Serine hydroxymethyltransferase (Serine methylase) (SHMT)	-3,8
HEAR2392	*cysI*	Sulfite reductase	-2,9
HEAR2394	*cysH*	Phosphoadenosine phosphosulfate reductase (PAPS reductase, thioredoxin dependent) (PAdoPS reductase) (3'-phosphoadenylylsulfate reductase) (PAPS sulfotransferase)	-2,6
HEAR2395	*cysD*	Sulfate adenylyltransferase subunit 2 (Sulfate adenylate transferase) (SAT) (ATP-sulfurylase small subunit)	-3,1
HEAR2396	*cysN*	Sulfate adenylyltransferase subunit 1 (Sulfate adenylate transferase) (SAT) (ATP-sulfurylase large subunit)	-3,4
HEAR2952	*metK*	S-adenosylmethionine synthetase (Methionine adenosyltransferase) (AdoMet synthetase) (MAT)	-2,4
Energy metabolism (14,4%)			
HEAR1086	*edd*	6-phosphogluconate dehydratase	-2,1
HEAR1813	*nuoN*	NADH-ubiquinone oxidoreductase, chain N	-3,4
HEAR1814	*nuoM*	NADH-quinone oxidoreductase subunit M	-4
HEAR1815	*nuoL*	NADH-quinone oxidoreductase subunit L (NADH dehydrogenase I subunit L) (NDH-1 subunit L)	-4,3
HEAR1816	*nuoK*	NADH-quinone oxidoreductase subunit K (NADH dehydrogenase I subunit K) (NDH-1 subunit K)	-4,3
HEAR1817	*nuoJ*	NADH-quinone oxidoreductase subunit J (NADH dehydrogenase I subunit J) (NDH-1 subunit J)	-2,3
HEAR1818	*nuoI*	NADH-quinone oxidoreductase subunit I (NADH dehydrogenase I subunit I) (NDH-1 subunit I)	-3,7
HEAR1819	*nuoH*	NADH-quinone oxidoreductase subunit H (NADH dehydrogenase I subunit H) (NDH-1 subunit H)	-2,6
HEAR1820	*nuoG*	NADH-quinone oxidoreductase subunit G (NADH dehydrogenase I subunit G) (NDH-1 subunit G)	-3,8
HEAR1821	*nuoF*	NADH-quinone oxidoreductase subunit F (NADH dehydrogenase I subunit F) (NDH-1 subunit F)	-3,3
HEAR1822	*nuoE*	NADH-quinone oxidoreductase subunit E (NADH	-3,2

		dehydrogenase I subunit E) (NDH-1 subunit E)	
HEAR1823	*nuoD*	NADH-ubiquinone oxidoreductase D subunit (NADH dehydrogenase subunit D)	-3,2
HEAR1824	*nuoC*	NADH-quinone oxidoreductase chain C (NADH dehydrogenase I, chain C) (NDH-1, chain C)	-3
HEAR1825	*nuoB*	NADH-quinone oxidoreductase subunit B (NADH dehydrogenase I subunit B) (NDH-1 subunit B)	-2,8
HEAR2748	*rubA2*	Rubredoxin	-2,1
HEAR3404	*atpC*	ATP synthase epsilon chain (ATP synthase F1 sector epsilon subunit)	-3,7
HEAR3405	*atpD*	ATP synthase subunit beta (ATPase subunit beta) (ATP synthase F1 sector subunit beta)	-3,2
HEAR3406	*atpG*	ATP synthase gamma chain (ATP synthase F1 sector gamma subunit)	-3,1
HEAR3407	*atpA*	ATP synthase subunit alpha (ATPase subunit alpha) (ATP synthase F1 sector subunit alpha)	-3,2
HEAR3408	*atpH*	ATP synthase delta chain AtpH	-2,7
HEAR3409	*atpF*	ATP synthase F0, B chain	-2,9
HEAR3410	*atpE*	Mambrane bound ATP synthase	-2,3
HEAR3411	*atpB*	ATP synthase F0, A chain	-2,1
DNA metabolism (0,6%)			
HEAR2573	*ihfB*	Integration host factor subunit beta (IHF-beta)	-7,7
Fatty acid and phospholipid metabolism (0,6%)			
HEAR2078	*plsX*	Fatty acid/phospholipid synthesis protein PlsX	-2,1
**PROTEIN FATE (0,6%)**			
HEAR0844	*lspAB*	Lipoprotein signal peptidase (Prolipoprotein signal peptidase) (Signal peptidase II) (SPase II)	-2,7
**TRANSCRIPTION (2,5%)**			
HEAR3141	*rpoA*	RNA polymerase, alpha subunit	-5,2
HEAR3172	*rpoC*	DNA-directed RNA polymerase subunit beta’ (RNAP subunit beta’) (Transcriptase subunit beta’) (RNA polymerase subunit beta’)	-3,3
HEAR3173	*rpoB*	DNA-directed RNA polymerase subunit beta (RNAP subunit beta) (Transcriptase subunit beta) (RNA polymerase subunit beta)	-3,7
HEAR3178	*nusG*	Transcription antitermination protein NusG	-4,2
**CELLULAR PROCESSES (1,9%)**			
HEAR0316		Bacterioferritin bfr (cytochrome b 557.5)	-2,2
HEAR2161	*priB*	Primosomal replication protein N	-5
HEAR2785		Conserved hypothetical protein, utative GTPase	-2,1
