# Supplementary material for: Multiple controls affect arsenite oxidase gene expression in Herminiimonas arsenicoxydans
Source: BMC Microbiol. 2010 Feb 18;10:53. doi: 10.1186/1471-2180-10-53 (PMC2848651; doi:10.1186/1471-2180-10-53)
Supplement: Additional file 2 — Supplemental table S2. Oligonucleotides used in the study. A. Identification of transposon insertion sites in H. arsenicoxydans mutants. B. Quantitative RT-PCR. [file 1471-2180-10-53-S2.PDF]

Additional material

Supplemental table S2. Oligonucleotides used in the study.

A. Identification of transposon insertion sites in *H. arsenicoxydans* mutants**.**

**Corresponding gene name**	**Primer name**	**Primer sequence (5'-3')**
aoxB	aoxB forward	AAATGACAGCATCTGGTGG
	aoxB reverse	CCGTCAATCCAACATTCTG
aoxS	aoxS forward	TCTTCCTGGTGACTTACTA
	aoxS reverse	CAAATTGACTCACCCATGA
aoxR	aoxR forward	ATTAATGGACGAGGTACCG
	aoxR reverse	CGACTACTAGAGAAGTAGC
modC	modC forward	CTCTGCAATATCTGAACGG
	modC reverse	ACATTGAATCCAACCGGAC
modB	modB forward	GAGCAAAGGCAAAGACAAC
	modB reverse	GTCCGGTTGGATTCAATGT
dnaJ	dnaJ forward	GAGCCCAGCATAGAAGCTT
	dnaJ reverse	TTGTCTGAAGTCGCAAGAC
rpoN	rpoN forward	AGCAATCCCTGCAATTACGCG
	rpoN reverse	AATGGCTTGGGTGGTATACAG

B. Quantitative RT-PCR.

**Primer name**	**Primers sequence (5'-3')**
HEAR0118 forward	GATCAGCTCGGGATTGATGTAG
HEAR0118 reverse	GCATGAACAGGTGATCGTCATC
HEAR0478 forward	CCCACGTACCTTTGTAGTAAG
HEAR0478 reverse	GACGTGGTGGAAGTGTTTAATG
HEAR0482 forward	CAGGTCAATATCGTGTGCTC
HEAR0482 reverse	CAACCCGTTAGGTGGAATGC
HEAR0483 forward	GTGCGCTACGATGAAGTAAG
HEAR0483 reverse	GCTTACTGCGGGAAGTTCAC
HEAR2646 forward	GCACGCCCTTAATACCCTTG
HEAR2646 reverse	CGACTTGCACTGCGAGATTC
HEAR3109 forward	GCAGCAGGTTCAATGCAATC
HEAR3109 reverse	GCGAACACTTGCTGGAACAG
HEAR2922 forward	GGCATTCCTCGACTCGTTTC
HEAR2922 reverse	GGAGCCAGTCCCTTTGTTTC
